# Suprasternal dermoid sinus: A case report

**DOI:** 10.1016/j.ijscr.2024.109785

**Published:** 2024-05-22

**Authors:** Tarek Abdelazeem Sabra, Ahmed Amgad Mohamed, Marwa T. Hussien, Sarah Magdy Abdelmohsen

**Affiliations:** a Pediatric Surgery Department, Department of General Surgery, Assiut University, Egypt; b Faculty of Medicine, Helwan University, Cairo, Egypt; c Medical Research Group of Egypt, Negida Academy, Arlington, MA, USA; d South Egypt Cancer Institute, Assiut University, Egypt; e Pediatric Surgery Department, Department of General Surgery, Aswan University, Aswan, Egypt

**Keywords:** Congenital, Dermoid sinus, Neck anomaly, Sinus excision, Infants, Case report

## Abstract

**Introduction:**

Congenital anomalies in the neck region, such as dermoid sinuses, pose diagnostic challenges in pediatrics. Surgical excisions are vital to prevent complications.

**Presentation of case:**

A 7-month-old male infant had presented with a congenital suprasternal dermoid sinus, which had been evident since birth. Imaging confirmed the diagnosis, prompting surgical intervention under general anesthesia. A delicate excision was performed, guided by a methylene blue dye injection, followed by histopathological confirmation.

**Discussion:**

Dermoid sinuses typically manifest as cutaneous pits or sinus tracts, with a left-sided predominance and a female predilection. An accurate diagnosis relies on clinical examination and imaging studies to delineate the anomaly. Surgical excision remains crucial to prevent recurrence and complications.

**Conclusion:**

This case reaffirms the necessity of prompt and accurate diagnosis followed by surgical intervention for managing congenital dermoid sinuses. Ongoing research and collaborative studies are needed to further refine management strategies and improve outcomes for patients with these anomalies, particularly when presenting in atypical locations.

## Highlights

• Congenital suprasternal dermoid sinus: An uncommon midline cervical anomaly in pediatric patients.
• Importance of accurate diagnosis: Using ultrasound and MRI to confirm no commuication with the underlying structure communication.
• Successful surgical intervention: Delicate excision under general anesthesia to prevent recurrence and complications.
• Timing of surgical intervention: Consider delaying excision until 2–3 years old to minimize anesthesia risks in asymptomatic cases.

## 1 Introduction

Congenital anomalies affecting the neck region present diagnostic and therapeutic challenges in pediatric practice. Dermoid sinuses are thought to originate from the incomplete obliteration of pouches and clefts during embryogenesis. It forms a sinus tract lined with stratified squamous epithelium [1]. It is an uncommon anomaly with unclear incidence, forming a spectrum of developmental defects that need to be carefully evaluated and treated to avoid any potential complications. It may extend to deeper structures, posing a risk for recurrent infection and abscess formation if left untreated [2,3].

Herein, we report a case of a male infant with a congenital suprasternal dermoid sinus, which manifests as a hole at the base of the neck since birth. This case highlights the importance of accurate diagnosis and surgical intervention in managing congenital dermoid sinuses to prevent complications and ensure optimal outcomes. This manuscript was prepared following the SCARE guidelines [4].

## 2 Presentation of case

A 7-month-old male infant has presented with a hole at the bottom of his neck since birth. There were no other complaints reported by the parents, and no associated discharge or abnormalities were noted.

**Fig. 1:**
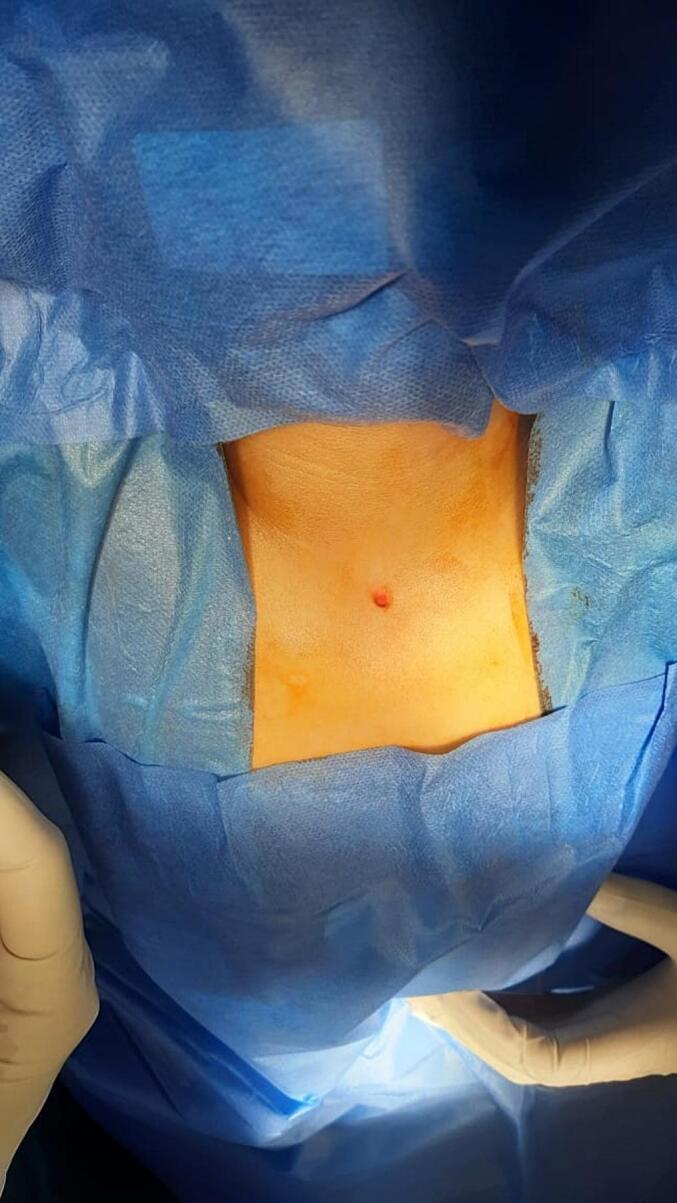
Sinus tract in the suprasternal region.

Upon examination, a subcutaneous sinus tract measuring more than 1 cm in depth was observed in the suprasternal region (Fig. 1). No signs of inflammation or discharge were observed. Initial ultrasound imaging was followed by an MRI with contrast, revealing no communication with the underlying structures.

**Fig. 2:**
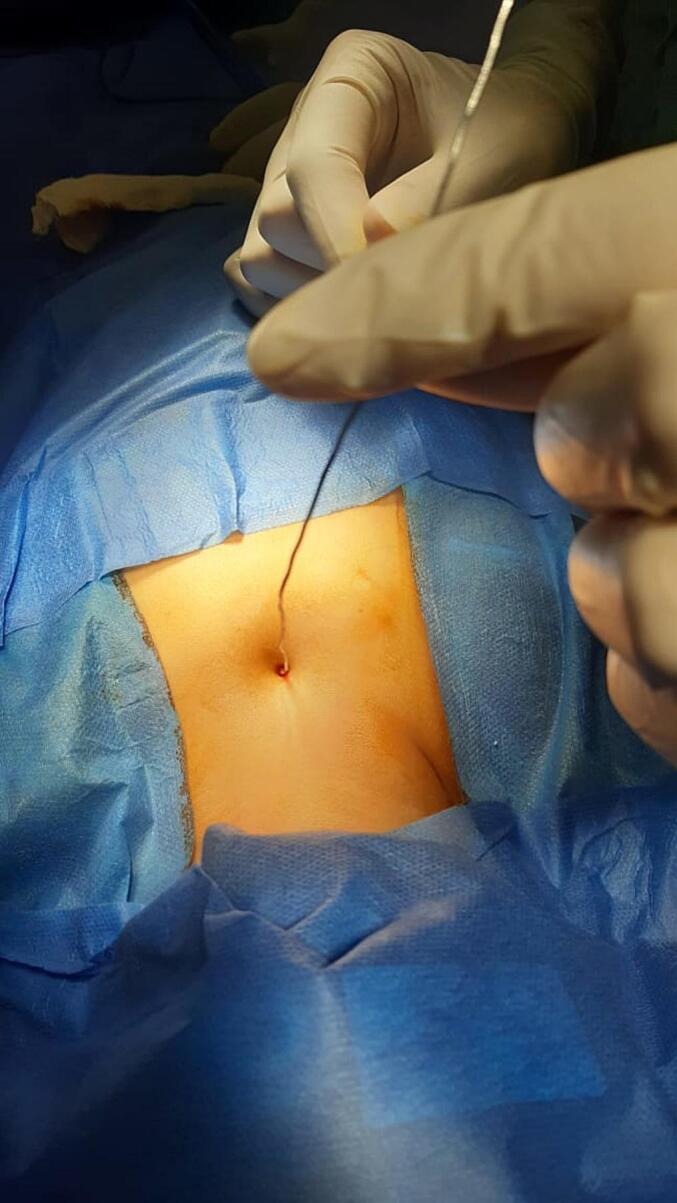
A blind needle was inserted in the sinus.

The patient underwent surgery under general anesthesia, during which the lesion was sterilized. A small amount of methylene blue dye was injected for identification. A blind needle was inserted (Fig. 2), followed by a circular incision around the sinus tract opening. The skin and a portion of the subcutaneous tissue were then removed. The edges were held by non-toothed forceps, and a careful dissection was performed to trace the tract to its blind end.

Approximately 0.25 cm of tissue was excised from all directions, and the subcutaneous tissue was closed with Vicryl 3-0 sutures, followed by skin closure with Proline 4-0 sutures.

**Fig. 3:**
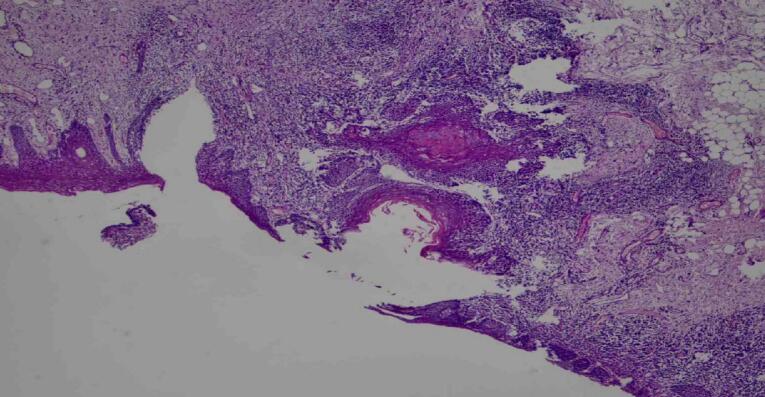
Histopathological examination showed keratinized squamous epithelium with focal erosion and the underlying dermis infiltrated by dense mixture of acute and chronic inflammatory cells (×10).

Pathological examination of the excised tissue revealed a sinus lined by bland-looking keratinized squamous epithelium with focal erosion, characteristic of a dermoid sinus, with infiltration of the dermis by a dense mixture of acute and chronic inflammatory cells (Fig. 3).

The follow-up period for this patient extended over several intervals, including 2 weeks, and 1, 3, and 6 months post-operatively. Throughout these evaluations, the patient displayed excellent healing without any signs of infection, recurrence, or other complications. The surgical site appeared well-healed, and the functionality of the surrounding tissue remained intact.

## 3 Discussion

In 1994, Matsunaga et al. were the first to describe the congenital dermoid sinus as a congenital dermoid fistula [5]. Shin et al. considered identifying the anomaly as a “sinus” rather than a “fistula” due to its pathological characteristics as a blind-end sinus [6]. Congenital dermoid sinus typically manifests as a cutaneous pit, sinus, or infected mass. In 2023, Alenezi conducted a comprehensive review of the literature regarding similar cases, shedding light on the evolution of different presenting symptoms and diagnostic trends [1]. Research indicates a notable left-sided predominance, with 92.3 % of cases occurring on the left, and a female dominance, accounting for 69.2 % of patients. The reasons behind these trends remain unclear. While present from birth, symptoms often arise after signs of infection develop. On average, the condition is diagnosed around 19.7 months of age [6].

The accurate diagnosis of these sinuses typically necessitates an integrated approach that includes both clinical examination and imaging studies [1,7,8]. Ultrasound and magnetic resonance imaging (MRI) are commonly employed for this purpose [1,7,8]. While MRI is considered the gold standard for imaging dermoid sinuses due to its superior ability to delineate soft tissue structures and produce detailed visualizations of the sinus tract and rule out associated anomalies, sometimes ultrasound only may be helpful [1,7,8].

Differential diagnosis of suprasternal or cervical sinuses is essential for identifying their specific origins and directing appropriate treatment [7]. Branchial cleft cysts and thyroglossal duct cysts are also significant etiological factors in the development of such sinuses [7]. Branchial cleft cysts, which are generally positioned along the lateral aspect of the neck, frequently lead to sinus formation due to anomalies in embryonic development [8]. Conversely, thyroglossal duct cysts, located centrally along the neck's midline, have a high propensity for creating sinuses, a condition exacerbated by their dynamic movement during swallowing and tongue protrusion [9].

Congenital dermoid sinuses present significant management challenges due to the potential for recurrent infections and abscess formation [10]. These complications can lead to extensive tissue damage if not adequately addressed [10]. A notable risk associated with these sinuses is the possibility of fistulization into internal structures, which may result in severe infections spreading along the sinus tract [10]. Chronic infections frequently result in the formation of fibrotic scar tissue, complicating surgical removal and adversely affecting cosmetic outcomes, especially in highly visible areas such as the neck [10]. The development of dense scar tissue may require more complex and invasive surgical approaches, increasing the risk of damage to nearby critical structures [10].

Surgical excision remains the cornerstone of treatment for dermoid sinuses to prevent recurrence and complications [5,11,12]. Additionally, alternative methods for treating sinuses have been explored, such as the use of Ethanolamine Oleate (EO) to induce sclerosis and Fibrin Glue to promote closure [13,14]. These techniques have shown efficacy in managing smaller sinus tracts that do not exceed 1 cm in diameter [13,14]. However, it is important to note that these approaches are still under investigation and may carry a risk of recurrence [13,14].

The successful management of this case underscores the importance of careful evaluation and treatment of congenital cervical anomalies. While early recognition, accurate diagnosis, and timely intervention are crucial to optimizing outcomes, our findings suggest that if asymptomatic, excision procedures can be delayed until children are 2–3 years old, when anesthesia risks are lower. However, in this case, the surgery was performed earlier because the patient lived in a rural area where medical access might be delayed, and complications could arise. This highlights the need to adjust treatment plans based on each patient's situation to ensure safety and effectiveness.

## 4 Conclusion

This case underscores the critical need for accurate identification and surgical management of midline cervical anomalies to achieve favorable outcomes. It also highlights the potential for complications if such conditions are left untreated. Further research and collaborative efforts are essential to deepen our understanding of the pathogenesis of dermoid sinuses and to refine the strategies for managing these conditions, especially when they occur in unusual locations. Such efforts will help optimize treatment protocols and improve patient care in similar cases.

## Ethical approval and consent from the participant

Approved.

## Consent for publication

Written informed consent was obtained from the patient's parents for publication of this case. A copy of the written consent is available for review by the Editor-in-Chief of this journal on request.

## Funding

None.

## CRediT authorship contribution statement

TAS The main surgeon. AAM Wrote the manuscript, edited it, and studied the analysis. MTH The pathologist. SMA Data collection, follow-up of the patients, study design, and final reviewer.

## Guarantor

Sarah Magdy Abdelmohsen

## Declaration of competing interest

The authors declare that they have no known competing financial interests or personal relationships that could have appeared to influence the work reported in this paper.

## Data Availability

Available when the editor requests.
